# Eyes on Development: Exploring the Impact of Early Life Nutrition Exposures on Child Cognition Through Eye-Tracking Assessment

**DOI:** 10.1093/nutrit/nuaf235

**Published:** 2025-12-06

**Authors:** Davrina Rianda, Ulrik N Mjaaseth, Michaela C DeBolt, Rina Agustina, Lisa M Oakes, Christine P Stewart

**Affiliations:** Department of Nutrition, University of California, Davis, CA 95616, United States; Institute for Global Nutrition, University of California, Davis, CA 95616, United States; Human Nutrition Research Center, Indonesian Medical Education and Research Institute, Faculty of Medicine, Universitas Indonesia, Jakarta 10430, Indonesia; Department of Nutrition, University of California, Davis, CA 95616, United States; Center for Mind and Brain, University of California at Davis, Davis, CA 95616, United States; Department of Psychology, University of California at Davis, Davis, CA 95616, United States; Human Nutrition Research Center, Indonesian Medical Education and Research Institute, Faculty of Medicine, Universitas Indonesia, Jakarta 10430, Indonesia; Department of Nutrition, Faculty of Medicine, Universitas Indonesia—Dr Cipto Mangunkusumo General Hospital, Jakarta 10430, Indonesia; Center for Mind and Brain, University of California at Davis, Davis, CA 95616, United States; Department of Psychology, University of California at Davis, Davis, CA 95616, United States; Department of Nutrition, University of California, Davis, CA 95616, United States; Institute for Global Nutrition, University of California, Davis, CA 95616, United States

**Keywords:** eye-tracking, child nutrition, maternal nutrition, cognitive development

## Abstract

**Background:**

The effects of certain nutrients on cognition in early life are hypothesized to be domain specific. Previous studies have examined the associations between child nutrition and cognitive, motor, or socio-emotional development, but the assessment methods are often insensitive to the specific functional domains affected by nutrition.

**Objective:**

This scoping review had 2 objectives: (1) to synthesize evidence on the impact of early life nutrition exposures on child cognition evaluated using eye-tracking assessment and (2) to describe the use of eye trackers for child cognitive assessment and to provide recommendations for eye-tracking implementation in future research.

**Methods:**

A systematic search was performed using the databases PubMed, Embase, and Food Science and Technology Abstracts, from database inception to April 2024. Additionally, we hand searched reference lists of eligible studies to identify additional relevant publications. We included studies of any nutrition exposures (intervention or assessment) involving pregnant or lactating mothers or children aged 0-5 years, with child cognitive outcomes assessed using eye-tracking assessments. The review was conducted following the PRISMA-ScR checklist.

**Results:**

A total of 1140 article titles and abstracts were screened, with 9 articles included in this review (3 randomized controlled trials, 5 prospective cohort studies, and 1 cross-sectional and prospective cohort study nested in a trial). The majority of the studies were conducted in low- and middle-income countries. Despite the limited number of nutrition studies using eye-tracking assessments, 5 of the 9 studies demonstrated significant relationships between nutrition exposures and attention and memory outcomes. Eye-tracking assessments were also found to be feasible across multiple settings.

**Conclusion:**

Eye-tracking assessment is a feasible and promising method for evaluating the effects of early life nutrition exposures on attention and memory outcomes across settings. However, the selection of eye-tracking–based tasks should be tailored to the type of nutrition exposures, the desired long-term outcomes, and the feasibility of proper training for local staff.

## INTRODUCTION

Cognitive development in early life lays the foundation for life-long capabilities, with the period from birth to 3 years of age considered the most critical.^1–3^ During this period, infants and young children experience rapid development of their cognitive abilities (eg, attention and memory), many of which are important for later academic performance.^4^^,^^5^ Nutrition in early life, encompassing both maternal and child nutrition, is a pivotal biomedical factor impacting cognition.^6^^,^^7^

Adequate nutrient intake is essential for infants and young children to support the structural development of their brains and enable effective engagement with their environment.^8^ Moreover, maternal nutrition during pregnancy represents an additional sensitive period during which nutrition interventions could impact fetal neurodevelopment, when neurogenesis and synaptogenesis occur.^9^ Several nutrients have been linked with brain development in children,^8^ including omega-3 fatty acids,^10^ iron,^11^ zinc,^12^ folic acid and choline,^13^ vitamin D,^14^ vitamin B6, and vitamin B12.^15^

Notably, the effects of certain nutrition exposures on cognitive development are hypothesized to be domain specific.^16^ Previous studies have examined the associations between child nutrition and cognitive, motor, or socio-emotional development. However, the assessment methods are often insensitive to the specific functional domains affected by malnutrition. In a systematic review of the effects of nutrition interventions on cognitive development among preschool-age children, the majority of the studies used global developmental tests (eg, the Wechsler Pre-school and Primary Scale of Intelligence [WPPSI] and Bayley Scales of Infant and Toddler Development 3rd edition [Bayley-III]).^17^ These tests are based on normative developmental milestones and assume the presence of a single underlying factor that accounts for all individual differences in cognitive status. Thus, the results of these tests are typically quantified as single, overall composite scores. Rather than affecting global outcomes, the effects of a given nutrition intervention might be specific to certain developmental domains influenced by the role of nutrients in specific biological pathways. Therefore, more precise results may be obtained using specific cognitive tasks. For instance, Cao et al^18^ found that docosahexaenoic acid (DHA) omega-3 is primarily accumulated in the hippocampus of rats and the parietal lobe of the brain and is associated with visual attention and working memory. In humans, a study by Rodrigue et al^19^ demonstrated that iron also accumulates in the hippocampus and correlates with memory performance. This finding is consistent with the findings of another study showing lower recognition memory among neonates with iron deficiency.^20^ Thus, the effect of such nutrients on development may be more clearly observed if the assessment targets those cognitive systems.

There has been growing interest in utilizing eye-tracking technology in nutrition research to measure specific areas of cognitive development. This technology enables the automatic measurement in children of eye fixation on stimuli, which provides insights into how long they look, where they look, and how they visually scan the stimuli.^21^ These looking behaviors can then be used to evaluate specific domains of child cognition, including attention, memory, language, social cognition, executive function, and predictive processing.^22–26^ Importantly, eye-tracking–based tasks reduce the necessity to have observers manually measure looking time. Stimuli presented during the tasks can also be adapted to local contexts, enabling cross-cultural applications.^27^

In this scoping review we aimed to synthesize existing research assessing the relation between maternal and child nutrition exposures in early life and child cognitive development as measured by eye tracking assessments. We began with a literature review describing the history and application of eye-tracking technology in assessing cognitive development in children, including its biological rationale and relevant developmental domains. We then present findings from the scoping review of nutrition studies on child cognition utilizing eye-tracking methods. Finally, we provide recommendations for future research implementing eye-tracking assessments to evaluate the impact of nutrition on child cognitive development.

### History of Eye-Tracking Assessment

Vision is one of the first senses to develop and is crucial for allowing infants to begin gathering information about the world around them. Even as newborns, humans selectively direct their gaze toward particular objects and events. Before language and advanced motor skills emerge, measuring looking behavior provides an indirect way to assess brain development, including cognition. Variations in looking behavior among children may reflect differences in their cognitive growth. For example, variations in visual attention, as reflected in how children direct their gaze, can indicate differences in their ability to focus on the meaningful parts of a visual stimulus.

Pioneering work using looking behavior in children to make inferences on their cognitive function emerged around the 1960s and 1970s.^28^^,^^29^ This early work revealed important characteristics of visual attention in infants. For example, infants look longer at novel or more complex stimuli than at familiar or less complex stimuli.^30–33^ Moreover, preferences for visual novelty in infants indicates early visual recognition memory and has been associated with later intelligence quotient (IQ) scores.^34^ Because the development of visual attention milestones varies among individuals, visual attention serves as a marker for later outcomes, including mathematics and literacy skills, as well as the potential for a diagnosis of autism spectrum disorder.^35^^,^^36^

Historically, looking behavior in children has been recorded via observations by a human coder through a peephole or from offline coding of films or videotapes; these methods are still widely used. Coders are trained to assess the direction of the gaze in infants when it falls within regions of a stimulus field. However, this method has challenges. First, it is more difficult to assess the vertical gaze position compared to the horizontal position.^37^ As a result, this type of observation is primarily suitable for measuring visual attention in paradigms that do not require precise measurement of vertical gaze, such as single-stimulus habituation paradigms or the 2-stimulus preferential-looking paradigm (when the items are presented side by side). More complex attention patterns, such as selectively attending to the eyes in a face, are challenging to evaluate. Additionally, assessing quantitative characteristics of eye movements, such as the timing of saccades and smooth pursuit, is not feasible using human observers. Last, although trained coders achieve high reliability ratings, and video recordings allow more detailed temporal resolution, the processes of determining eye position through these recordings and analyzing the data are often extremely time -consuming.

Objective evaluation of eye movements emerged when 2 automated eye-tracking techniques were developed.^37^ Electro-oculography (EOG) is a technique based on the gradient of metabolic activity between the front and back of the eyeball. However, a fundamental problem with this technique is that it requires a pair of electrodes to be placed near the eyes, which is often not feasible for infants. Metabolic activity also varies over time and may cause the EOG signal to drift without any gaze change. Another technique, corneal-reflection photography, was developed based on the occurrence of corneal reflection (CR) when a small point of light is directed to the eye. Although this technique can provide detailed measurements of changes in gaze, CR requires the head of the child being tested to be fixed in a single position. A video camera with a close-up lens that fills the video frame with the eye, focusing on the pupil, is used in CR photography. Any movement that causes the pupil to move outside the video frame results in a loss of usable data. To resolve this issue, eye-tracking systems require position sensing to monitor head movements. Early systems used a magnetic head tracker (MHT) receiver placed on the head of the infant and connected to an MHT control unit. More modern systems involve video solutions to monitor head movements. The development of the CR eye-tracking technique allowed children to move freely while maintaining high spatial and temporal accuracy of eye position recording.^38^ This recent technique is promising for use in nutrition studies across various settings, including remote or resource-constrained areas, when measuring cognition is an outcome.

### Cognitive Domains Evaluated Using Eye-Tracking Assessment

**Figure 1.**
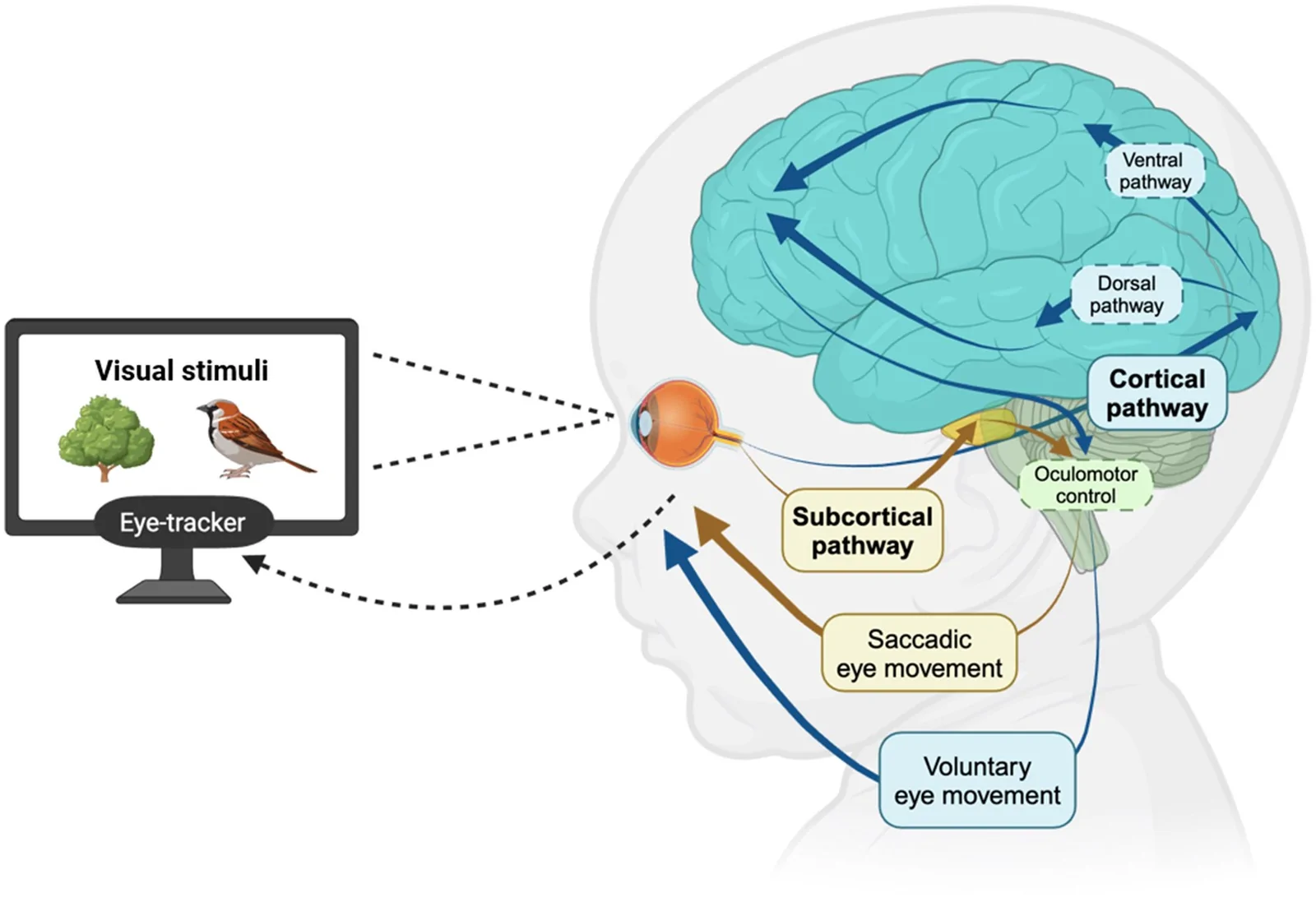
Mechanistic Pathway of Visual Processing Captured by Eye-Tracking Assessment

Eye-tracking assessments capture eye gaze, reflecting visual processing in the brain. The mechanistic pathway of visual processing assessed by an eye tracker is described in Figure 1. The retina of the child being tested receives stimuli displayed on the screen. Two visual processing pathways—subcortical and cortical—are activated from the retina. The subcortical pathway transmits visual input to the superior colliculus in the midbrain.^39^ The information is relayed to the basal ganglia. This method allows saccadic eye movements—quick, simultaneous shifts of both eyes that change the fixation point. These eye movements are important for a wide range of visual abilities, including tasks involving attentional cues.

The cortical pathways of visual processing pass visual input to the cortical areas via the primary visual cortex in the occipital lobe.^40^ From there, the input is transmitted via 2 pathways, the ventral visual pathway through the temporal lobe and the dorsal visual pathway through the parietal lobe.^40^^,^^41^ Visual information is processed in distinct ways—object recognition and identification occur via the ventral pathway while spatial awareness is processed via the dorsal pathway. These functions help children identify stimuli and then focus their attention on selective information when saccades are planned. Some stimuli trigger the recognition and recall of memories, leading to eye movements that differentiate between novel and familiar stimuli. Finally, voluntary eye movements are initiated as visual processing passes through several areas in the frontal lobe that are responsible for voluntary eye movements, including the frontal eye field, the supplementary eye field, and the dorsolateral prefrontal cortex.^39^^,^^42^^,^^47^ Thus, the looking behavior of a child in response to varying stimuli results from visual processing that reflects attention and interactions with other cognitive and perceptual systems, such as memory and learning. Eye-tracking procedures allow for the evaluation of these cognitive domains, including attention and memory.

Attention is a fundamental cognitive process involving the selection of relevant information and inhibition of irrelevant information. These selection and inhibition processes allow children to orient toward, shift between, and maintain focus on targets.^43^ Visual attention promotes information gathering and selection, which determine the targets for subsequent learning and influence developmental trajectories across various cognitive domains. Multiple functions are constructed within attention. Four attentional functions are considered crucial for the learning process. These functions can be assessed using eye-tracking methods: (1) orientation toward and away from objects at various spatial locations, (2) selective filtering of visual inputs, (3) processing visual inputs, and (4) maintaining focus on a target.^44^ However, these attentional functions are often closely intertwined in everyday life. Therefore, a task designed to enable evaluation of 1 function may also engage other attentional functions and/or cognitive domains. For instance, tasks used to measure the duration of attention to screen-based stimuli primarily assess the ability to maintain focus on a target. Yet, these tasks may also involve the attentional function of processing visual inputs, executive attention, and motivational factors (ie, the preference of a child for specific stimuli).

Another cognitive domain that can be assessed using eye-tracking methods is memory. Memory is a product of encoding, storing, and retrieving information, and therefore is tightly linked with learning.^43^ When children look at an object, they begin to form a memory representation; whether that memory is fleeting and stored in visual short-term memory or becomes consolidated as a long-term memory depends on several factors, including how long the child looks at the object. However, it is worth noting that fidelity of memory is also affected by attentional selectivity, which redirects infants to relevant objects, and perceptual discrimination, which is required for children to discriminate objects as different entities.

Most studies assessing visual memory among infants and young children are based on habituation/novelty preference, which first allows children to become familiar with, or form a memory of, an image and then compares their responses to novel and familiar images. As the memory of a child for an image grows, the child will exhibit increased looking behavior at a novel stimulus. Therefore, the appearance of a “novelty preference” indicates memory of a familiar image. Moreover, certain tasks might be useful for assessing functions related to specific brain areas. For example, a relational binding task that evaluates the ability of an infant ability to bind features (ie, identify objects by features and bind them to locations, learning both “what” and “where,” for example learning that one’s toys are in the family room). This ability is related to the further development of medial temporal lobe structures, including the parahippocampal cortex.^45^

### General Procedure of Eye-Tracking Assessment

Eye-tracking systems estimate a point of gaze and calculate aspects of eye movements from corneal and pupil reflections. When a light source reflects off the cornea, the reflection remains relatively stationary as the eye moves. This reflection is called the first Purkinje image. The gaze location can then be calculated by comparing the position of the first Purkinje image to the position of the center of the pupil.^38^ To link the angular rotations of the eye to known spatial positions on the screen, a short (∼20 s) calibration procedure is performed at the start of the eye-tracking assessment.^46^

When selecting the type of eye tracker, an important consideration is the sampling rate. Modern eye trackers have sampling rates ranging from 30 to 1200 Hz; earlier models had rates ranging from 60 to 120 Hz. A trade-off may occur between sampling rate and data quality (eg, lost samples, accuracy, precision). A lower sampling rate decreases temporal precision (eg, 16 ms at 60 Hz vs 8 ms at 120 Hz), but it eases the calibration process, making it easier for non-experts to track infants and therefore minimizes data loss from the analysis.^46^

**Figure 2.**
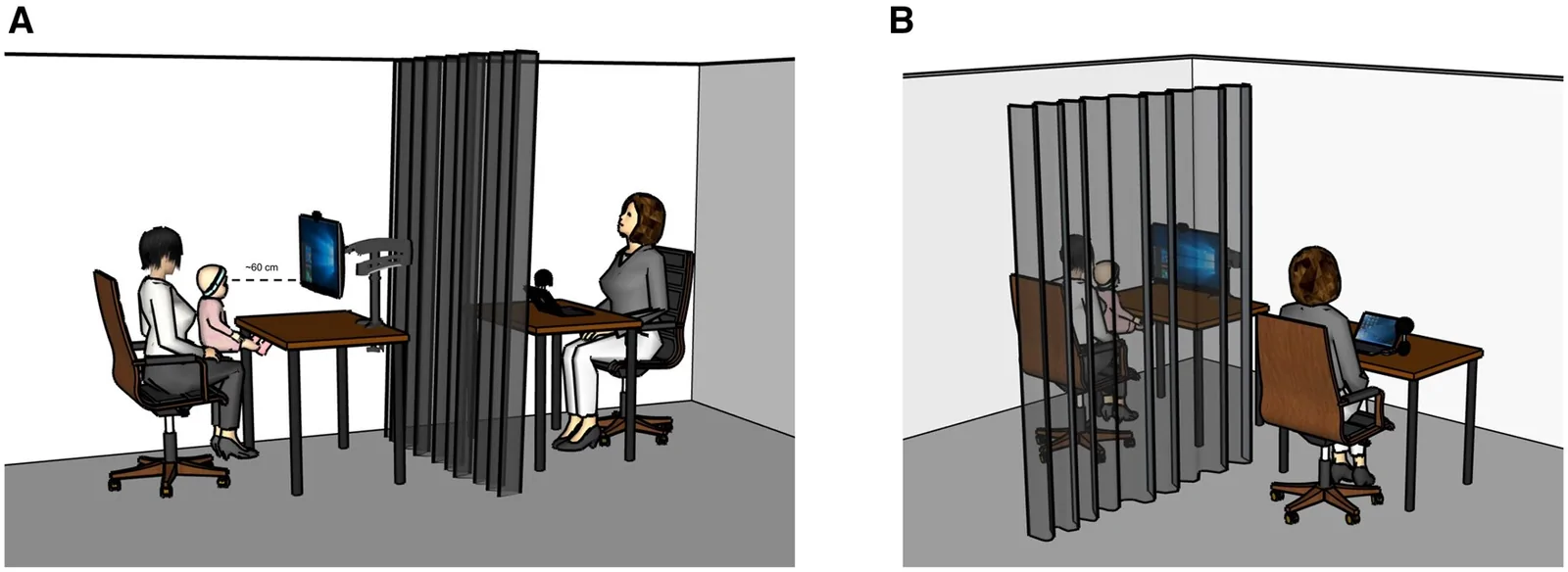
General Setup for Eye-Tracking Assessment: (A) Assessor facing the mother and infant, or (B) assessor sitting next to the mother and infant

Figure 2 illustrates the general setup for an eye-tracking assessment. The assessment should be conducted in a room or curtained booth with minimal distractions and a quiet environment. The testing area should have no windows because daylight contains some frequencies in the infrared spectrum. Artificial lighting in the room should be diffused to prevent reflections on the pupil, which could interfere with the eye tracker algorithm.

During the assessment, the child is positioned on their mother’s lap, in an infant carrier, or seated independently, facing the screen. The eye tracker is attached to the monitor and placed approximately 60 cm from the child’s eyes (the specific distance will depend on the make and model of the eye tracker). A monitor mount is often used to help adjust this distance. Additionally, a webcam records the child’s behavior, allowing the assessor to note any significant activities, such as when the infant becomes fussy.

A plain-colored divider is placed between the assessor and the mother and child. The assessor may either face the mother and child or sit beside them, as long as the position allows them to intervene when needed. For instance, if the child loses focus, the assessor can shake a rattle behind the monitor to redirect the child’s attention back to the screen. Therefore, adequate space behind the monitor is necessary during the assessment.

### Assessing Child Cognition Using Eye-Tracking Methods in Nutrition Studies

Utilizing eye-tracking methods is a promising approach with significant implications for understanding child development and nutrition. When evaluating the influences of nutrition exposures on child cognition, selecting appropriate eye-tracking–based tasks requires several considerations. Certain tasks have been validated and have demonstrated stronger associations with long-term outcomes. For example, although both methods assess memory, the visual paired comparison (VPC) task has shown larger associations with later intelligence quotient (IQ) than the habituation task.^47^^,^^48^

Importantly, assessments of infant looking behavior have shown large individual and developmental differences, suggesting that these methods might be more suitable for assessing group-level differences rather than individual changes.^47^ A study conducted among infants in Malawi demonstrated low test–retest correlations for eye-tracking results, even over a period as short as 2 months (ie, between 7 and 9 months of age).^49^ In general, looking measures in infancy that are separated at points close in time (a few days or weeks) tend to give moderate reliability (0.40-0.60), while those separated by a month or more showed lower estimates (0-0.20).^50^ This variation can be attributed to the different rates at which children develop, leading to increased within-person differences over time. Due to this high variability in eye-tracking results, ensuring a sufficient sample size is also crucial.

Assessing general cognitive development with eye-tracking methods is more sensitive in detecting subtler deficits or benefits from a nutrition intervention than using broader assessments of early neurodevelopment, such as the Bayley Scales of Infant and Toddler Development.^16^ Notably, some eye-tracking–based tasks are associated with specific brain regions, which may be advantageous for evaluating nutrition interventions targeting particular cognitive areas. For instance, assessment of a nutrition intervention hypothesized to affect the parahippocampal cortex could be improved by using a relational binding task.^51^ Tasks involving preferential tracking of faces can be used to evaluate interventions that are hypothesized to affect the posterior temporal cortex.^52^ However, although eye-tracking–based tasks are designed to assess specific cognitive domains, most of them may also engage other domains. For instance, habituation/novelty preference tasks primarily assess memory but also require attentional engagement. Moreover, some tasks may involve activation across multiple brain regions. For example, tasks targeting spatial attention, such as the Infant Orienting with Attention (IOWA) task, engage frontal, prefrontal, and parietal cortical areas.^53^^,^^54^ Thus, these tasks are suitable even when the cognitive domains or brain regions influenced by specific nutrients are not yet identified.

One potential challenge for the use of eye-tracking methods is data loss. Enrolled children may not complete eye-tracking assessment due to loss to follow-up, refusal to participate, or failure to achieve successful calibration. Even among the children who are have undergone assessment, data loss can still occur if children move excessively or turn their heads away from the screen (eg, due to inconsolable crying, being too tired, or not being focused on the screen) or if the eye tracker fails to detect the pupil in the camera image.^55^^,^^56^ These issues can result in children having insufficient usable data for analysis. To the best of the authors’ knowledge, the typical rate of data loss in eye-tracking studies among infants and young children has not been systematically evaluated. However, 1 study examining eye-tracking data quality in infants 5 and 10 months old found that data loss was predicted by both participant age and assessor.^56^ Among 5-month-old infants, rates of data loss were higher, with a peak around 15%, and showed greater variability across assessors compared to 10-month-old infants. In nutrition studies, data loss is an issue particularly if exclusions are not random but are systematically related to the child’s nutritional status. For example, a nutrition intervention that reduces fussiness alongside improving attention and memory might result in more available data in the intervention group compared to control.

## METHODS

We used the Preferred Reporting Items for Systematic Reviews and Meta-Analyses extension for Scoping Reviews (PRISMA-ScR) Checklist to guide our scoping review process. The protocol for this review was registered with the Open Science Framework in April, 2024 (https://osf.io/fkab2/).

### Search Strategy

We performed a literature search using the PubMed, Embase, and Food Science and Technology Abstracts databases from inception to April 2024, when our protocol was registered. We used the search terms in **Table S1**, adapted for each bibliographic database and developed under the guidance of an experienced librarian at the University of California, Davis. To identify any additional relevant publications, we individually searched reference lists from eligible studies. Specific nutrients of interest defined as search terms were selected based on reviews by Nyaradi et al.,^57^ Prado and Dewey,^8^ and Cusick and Georgieff.^58^ Our search strategy used the following structure: *Population* AND *Intervention/Comparison* AND *Outcome*. All studies found based on the combined search across all databases were uploaded into Covidence (www.covidence.org) to remove duplicates and screen the titles and abstracts of the articles.

After removing duplicates, 2 authors (D.R., U.N.M.) independently performed title and abstract screening. We included studies of any nutrition exposures (intervention or assessment) involving mothers and/or children with outcomes on child cognition assessed using any eye-tracking methodology. We searched for any human observational studies or trials in pregnant or lactating mothers, infants (<1 year old), or young children (1-5 years old), in which eye-tracking methods were used to assess child cognition at any point after supplementation. We included any types of nutrition exposure or indicator of status (eg, serum ferritin concentration) and nutrition interventions, including foods, supplements, or dietary recommendations to consume nutrient-rich foods. Systematic reviews of effects of nutrition intervention on child development were included to identify potential additional studies. Unpublished studies were not included, and studies were restricted to those published in English only.

### Full-Text Review and Data Extraction

To determine the eligibility of the identified studies, 1 author (D.R.) reviewed all full-text versions of the potentially relevant studies, and 33.3% of the studies (*n* = 62 studies) were randomly selected for a double review by another author (U.N.M.). Any uncertainties during this full-text eligibility assessment were discussed with other authors. We contacted the corresponding authors of studies with undisclosed data, requiring clarification, or published as study protocols to determine whether outcome data had since been published.

We charted data from the included studies using a pre-designed form. One author (D.R.) performed data charting for all studies, with 33.3% (*n* = 3 studies) of randomly selected studies doubled-charted by another author (U.N.M.). These data included (1) first author and year, (2) study design, (3) study population and sample size, (4) study location, (5) type and duration of nutrition exposure, (6) type of eye-tracking–-based task as the outcome, and (7) findings. We considered critical appraisal of the studies unnecessary for this scoping review. A narrative synthesis and overview tables were provided to summarize all included studies.^59^ We created figures using BioRender (www.biorender.com) for the mechanistic pathway of visual processing (Figure 1) and SketchUp (www.sketchup.com) for the general setup of eye-tracking assessment (Figure 2).

## RESULTS

**Figure 3.**
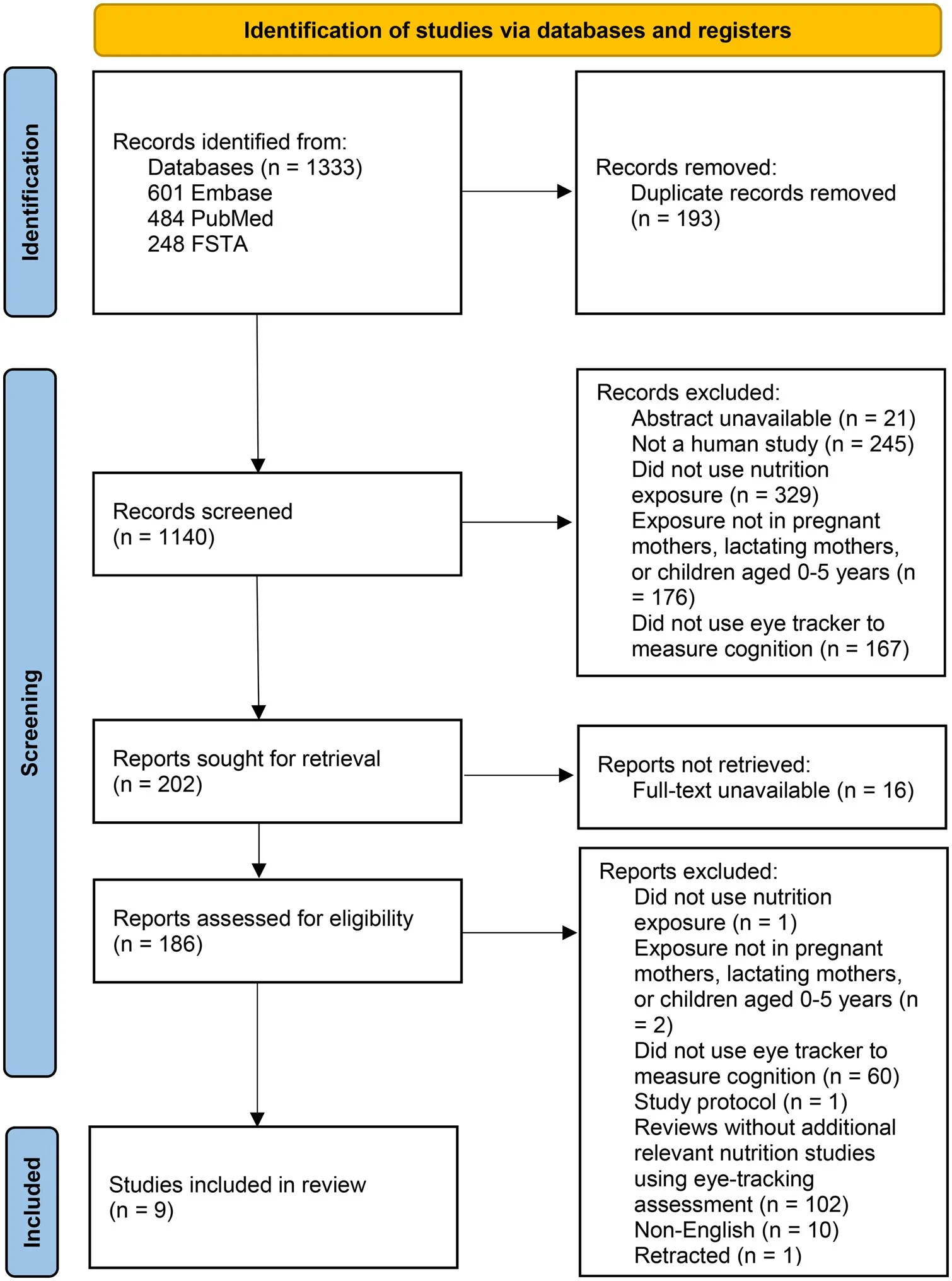
PRISMA Flow Diagram

We screened a total of 1140 article titles and abstracts, and 186 full-text articles were retrieved and assessed for eligibility, resulting in 9 articles included in this review (Figure 3). The primary reason for excluding empirical articles was the absence of an eye-tracking assessment to measure cognition (*n* = 60 articles), and reviews (*n* = 102 articles) were excluded when they did not provide additional relevant nutrition studies that used eye-tracking assessment. Of the 9 articles included, 3 were controlled trials,^60–62^ 5 were prospective cohort studies,^49^^,^^55^^,^^63–65^ and 1 was a cross-sectional and prospective cohort study nested in a trial.^66^ Only 2 studies were conducted in high-income countries^63^^,^^64^ (ie, Singapore and England); the remaining 7 studies were conducted in low- and middle-income countries^49^^,^^55^^,^^60–62^^,^^65^^,^^66^ (ie, Malawi [*n* = 4], Sierra Leone [*n* = 1], South Africa [*n* = 1], and Gambia [*n* = 1]).

**Figure 4.**
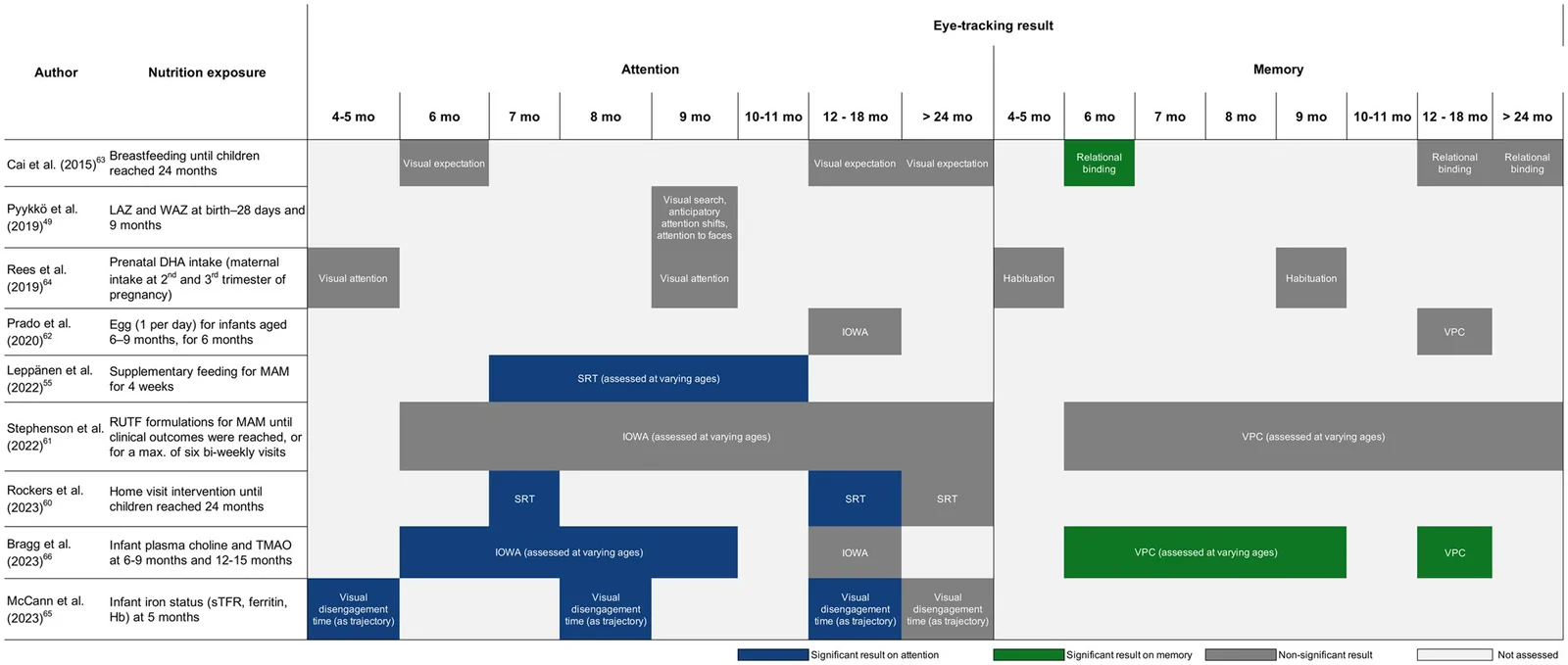
Summary of Relationships between Maternal and Child Nutrition Exposures and Eye-Tracking Outcomes in the Included Studies. Abbreviations: DHA, docosahexanoic acid; Hb, hemoglobin; IOWA, Infant Orienting with Attention; LAZ, length-for-age *z* score; MAM, moderate acute malnutrition; SRT, saccadic reaction time; sTfR, soluble transferrin receptor; TMAO, trimethylamine N‐oxide; VPC, visual paired comparison; WAZ, weight-for-age *z* score.

All 9 included studies involved varied nutrition exposures, with 2 reporting on the same study sample (**Table 1**).^49^^,^^55^^,^^60–66^ Eye-tracking procedures used in the included studies are described in Table 2.^49^^,^^55^^,^^60–66^ These procedures focus on evaluating 2 cognitive domains: attention and memory. Across 11 different eye-tracking–based tasks, only 3 were used in more than 1 study: saccadic reaction time (SRT), VPC, and IOWA tasks. Randomized controlled trials included in this review tested various nutrition interventions, consisting of eggs, ready-to-use therapeutic food (RUTF), formulation for moderate acute malnutrition (MAM), and home visit interventions that included child nutrition. Other nutrition exposures were assessed through prospective cohort and cross-sectional studies, which examined breastfeeding, length-for-age *z* scores (LAZ) and weight-for-age *z* scores (WAZs), prenatal DHA intake, supplementary feeding for MAM, plasma choline and trimethylamine N-oxide (TMAO), and iron status. Our literature search revealed that the earliest study on nutrition utilizing eye-tracking assessment was published in 2015.^63^ Of the 9 studies included, significant associations between maternal and child nutrition exposures and eye-tracking outcomes were demonstrated in 5 studies with varied types of exposures and eye-tracking–based tasks, as summarized in **Figure 4.**^49^^,^^55^^,^^60–66^

**Table 1.** Description of Included Studies

Reference	Study design	Country	**Study population for eye-tracking assessment** ^a^	Nutrition exposure	Duration	Eye-tracking–based tasks	Outcome
Cai et al. (2015)^63^	Prospective cohort study	Singapore	*n* pregnant women enrolled = 1247 *n* children born and enrolled = 1247 *n* at 1st visit (6 mo) = 473 (attending); 185 (analyzed) *n* at 2nd visit (18 mo) = 431 (attending); number of children included in the analysis not reported *n* at 3rd visit (24 mo) = 514 (attending); number of children included in the analysis not reported	Breastfeeding (duration and exclusivity)	From pregnancy until 24 mo	Memory Relational binding Attention Visual expectation	Better memory at 6 mo, indicated by greater preferential looking toward correctly matched items during early portions of relational binding task, among higher breastfeeding exposure No significant association between breastfeeding and visual expectation
Pyykkö et al. (2019)^49^	Prospective cohort study	Malawi	*n* infants enrolled = 444 *n* at 9 mo = 397 (assessed); 384 (analyzed)	LAZ and WAZ at enrollment (birth–28 d old) and 9 mo	From birth until 9 mo	Attention Visual search Anticipatory attention shifts Attention to faces	No significant correlations between any eye-tracking scores and LAZ or WAZ
Rees et al. (2019)^64^	Prospective cohort study	England	*n* pregnant women enrolled = 125 *n* children born and enrolled = 107 *n* at 1st visit (4.5 mo) = 101 (attending); number of children included in the analysis not reported *n* at 2nd visit (9 mo) = 94 (attending); number of children included in the analysis not reported	Prenatal DHA intake (maternal intake at 2nd and 3rd trimester of pregnancy)	From pregnancy until 9 mo	Memory Habituation Attention Visual attention	No significant differences in habituation and visual attention tasks based on prenatal DHA intake
Prado et al. (2020)^62^	Randomized controlled trial	Malawi	*n* infants enrolled = 331 intervention vs 329 control *n* = 282 intervention vs 290 control (assessed); 236 intervention vs 239 control (analyzed) Age: 6–9 mo	Egg (1 per day)	6 months	Memory VPC Attention IOWA	No significant effects of the intervention on VPC and IOWA tasks
Leppänen et al. (2022)^55^	Prospective cohort study nested in a cluster-randomized trial	Sierra Leone	*n* infants enrolled = 138 with MAM vs 60 age-matched control *n* = 135 MAM vs 56 control (assessed); 96 MAM vs 46 control (analyzed) Age: 7–11 mo	Supplementary feeding for infants in MAM group: Corn-soy blend plus with oil corn-soy-whey blend with oil super cereal plus with amylase, or ready-to-use supplementary food	Follow-up until 4 wk after intervention started	Attention SRT	Higher adjusted SRT at baseline and improvement in SRT 4 wk after intervention among MAM infants compared to controls
Stephenson et al. (2022)^61^	Randomized controlled trial	Malawi	*n* infants enrolled = 864 DHA-HO-RUTF vs 930 HO-RUTF vs 964 S-RUTF *n* subset of infants invited for eye-tracking assessment = 380 DHA-HO-RUTF vs 350 HO-RUTF vs 402 S-RUTF *n* = 248 DHA-HO-RUTF vs 244 HO-RUTF vs 297 S-RUTF (analyzed) Age: 6–59 mo with MAM	3 different RUTF formulations for MAM: DHA-HO-RUTF HO-RUTF S-RUTF	Until reaching a clinical outcome or for a maximum of 6 bi-weekly follow-up visits	Memory VPC Attention IOWA	No significant effects of the DHA-HO-RUTF or HO-RUTF, as compared with S-RUTF, on VPC and IOWA tasks
Rockers et al. (2023)^60^	Cluster-randomized controlled trial	South Africa	*n* infants enrolled = 182 intervention vs 134 control *n* at 1st visit (7 mo) = 182 intervention vs 134 control (assessed); 103 intervention vs 73 control (analyzed) *n* at 2nd visit (17 mo) = 184 intervention vs 132 control (assessed); 161 intervention vs 113 control (analyzed) *n* at 3rd visit (36 mo) = 164 intervention vs 120 control (assessed); 148 intervention vs 113 control (analyzed)	Home visit intervention integrated to community health workers operations, which included child nutrition	Intervention until children reached the age of 24 mo; follow-up until 36 mo	Attention SRT	Lower SRT at 7 mo and 17 mo among intervention group
Bragg et al. (2023)^66^	Cross-sectional and prospective cohort studies nested in a randomized controlled trial (Prado et al.^62^)	Malawi	*n* = 400 (analyzed) Age: 6–9 mo	Infant plasma choline and TMAO	6 months follow-up for longitudinal assessment	Memory VPC Attention IOWA	Significant association between higher plasma choline and slower IOWA response time Significant association between higher plasma choline and faster processing speed on VPC task Significant association between higher plasma TMAO at baseline and slower processing speed on VPC task at follow-up
McCann et al. (2023)^65^	Prospective cohort study	Gambia	*n* infants enrolled = 258 *n* at 1st visit (5 mo) = 195 (assessed); 150 (analyzed) *n* at 2nd visit (8 mo) = 188 (assessed); 143 (analyzed) *n* at 3rd visit (12 mo) = 187 (assessed); 134 (analyzed) *n* at 4th visit (18 mo) = 177 (assessed); 134 (analyzed) *n* at 5th visit (24 mo) = 164 (assessed); 106 (analyzed) *n* at 6th visit (3–5 yo) = 171 (assessed); 167 (analyzed)	Infant iron status at the age of 5 mo	From 5 mo to 5 yo	Attention Visual disengagement time	Significant differences on the trajectory of visual disengagement time between infants in highest and lowest terciles of sTfR No significant differences on the trajectory of visual disengagement time based on ferritin and Hb status

a N *assessed* includes all children who completed the eye-tracking assessment, regardless of whether the data were useable. *n analyzed* includes only children with sufficient useable data in at least one eye-tracking-based task. *n attending* refers to all children who attended the outcome assessment visit, regardless of whether they were assessed for eye-tracking, and is reported only when the study did not specify *n assessed*.

Abbreviations: DHA, docosahexanoic acid; DHA-HO-RUTF, ready-to-use therapeutic food made with HO peanuts and palm oil with added DHA; Hb, hemoglobin; HO, high oleic; HO-RUTF, ready-to-use therapeutic food made with high-oleic (HO) peanuts and palm oil without added DHA; IOWA, Infant Orienting with Attention; LAZ, length-for-age *z*-score; MAM, moderate acute malnutrition; SRT, saccadic reaction time; S-RUTF, standard RUTF; sTfR, soluble transferrin receptor; TMAO, trimethylamine N‐oxide; VPC, visual paired comparison; WAZ, weight-for-age *z* score.

**Table 2.** Overview of Eye-Tracking-Based Tasks in the Included Studies: Assessed Domain and Function, Procedures, and Outcome Indicators

Cognitive domain evaluated	Function assessed	Eye-tracking-based tasks	Procedures	Indicators	Reference citation
Memory	Binding in memory	Relational binding	Infant watched blocks of trials involving scenes and picture of toys. Each block consisted of study trial and test trial. Study trial: Infant watched 3 audiovisual scenes with a picture of a toy superimposed on each scene. Test trial: Infant watched 1 of 3 audiovisual scenes, with all 3 pictures of toys viewed previously superimposed on scene.	Proportion of time child spent looking at each picture to evaluate relational memory (child’s preference to look at correct match)	Cai et al. (2015)^63^
	Memory formation	Habituation	Infant watched repeated paired presentations of a familiar and novel stimulus. The side presentation of novel or familiar stimulus was random. Familiar stimuli: 4 yellow dots arranged in a shape of a square Novel stimuli: Patterns of 2, 3, 4, or 5 dots in either blue or red Trial was stopped if infant did not look at screen for 2 consecutive trials.	Look duration to familiar stimulus Number of trials required to be habituated (ie, a decrease to half of average looking time compared to initial looking time to familiar stimulus)	Rees et al. (2019)^64^
	Recognition memory	Visual paired comparison	Task consisted of 4 trials for 4 different pairs of local faces (ie, adult male-adult female, adult male-child male, adult female-child female, child male-child female). Each trial was composed of a familiarization followed by a recognition memory period. Familiarization period: 2 identical faces were presented on left and right side of screen. Recognition memory period: A new pair of faces were presented, consisting of same face shown in familiarization period (familiar stimulus) in one side and a novel face in other side. These stimuli were side reversed after first 5 s	Novelty preference score, or proportion of time child spent looking at novel face compared to previously seen face Peak look length Mean familiarization fixation	Prado et al. (2020)^62^, Stephenson. et al. (2022)^61^, Bragg. et al. (2023)^66^
Attention	Planning and learning patterns from visual inputs	Visual expectation	Baseline phase: Infant watched 18 movie clips presented in random locations on a screen Experimental phase: Infant watched similar clips presented in locations on screen that varied according to specific pattern (eg, left-left-right or right-right-left)	Saccadic reaction time Reaction time Average proportion of time spent looking at stimuli Proportion of trials when infants correctly anticipated Average duration of anticipation Average proportion of time spent looking at correct location in advance of stimulus appearing on screen	Cai et al. (2015)^63^
	Selective filtering of visual inputs	Visual search	Infant watched a presentation of an image of red apple with an “oh” sound on center of screen. Blank screen appeared, followed by reappearance of apple in randomly chosen location. Depending on experimental condition, apple was presented alone (*one-object condition*), among 4 or 8 distractors of 1 kind (*multiple-objects condition*), or among distractors of 2 kinds (*conjunction condition*). If infant fixated on apple within 4 s from start of trial, a reward sound was played while apple spun. Infant received total of 8 trials/condition in 2 sessions. Successful search was determined when gaze hit area of interest (apple) within predefined time limit.	Number of successful searches in *1 object* Number of successful searches in *multiple objects* Number of successful searches in *conjunction condition*	Pyykkö et al. (2019)^49^
	Planning and learning patterns from visual inputs	Anticipatory attention shifts	Infants were presented with a fixation stimulus (a pink pig face) in center of screen. After infant looked at fixation stimulus, an auditory cue and 2 visual placeholders (empty rectangles) were presented to left and right side of screen. The furthest edge of rectangles bordered edge of screen. If infant made a correct anticipatory saccade to placeholder where a salient visual stimulus was to be presented, or after 1000 ms had passed, an audiovisual reward appeared within rectangle. Reward appeared on 1 side and same side (left or right) during first 8 trials (*pre-switch*), then side was switched on last 8 trials (*post-switch*). Infant received a total of 16 pre-switch and 16 post-switch trials in 2 sessions.	Correct anticipation % in pre-switch % correct anticipation in post-switch	Pyykkö et al. (2019)^49^
	Maintaining focus on a target	Attention to faces	Infant watched a dynamic attention grabber on center of screen. After infant fixated on stimulus, 2 stimuli were presented with a 1000-ms onset asynchrony. The first stimulus was a picture of a nonface pattern, or a face displaying a happy or fearful expression, presented on center of screen. The 2nd stimulus was geometric shape, superimposed by still picture showing 1st frame of child-friendly cartoon animation. This stimulus was displayed laterally on left or right side and bordered screen edge. If infant shifted gaze to 2nd stimulus, still picture turned into dynamic cartoon. Infants received a total of 8 nonface trials and 8 face trials (4 happy, 4 fearful) in random order in 2 sessions.	Percentage of occurred shifts to non-face control stimuli Percentage of occurred shifts to lateral happy stimulus Percentage of shifts to lateral fearful stimulus Dwell time index on nonface control stimuli Dwell time index on faces	Pyykkö et al. (2019)^49^
	Maintaining focus on a target	Visual attention	Infant watched 4 consecutive trials presenting a stimulus, which was a circular, yellow, smiley face rotated around monitor on a circular path. On each trial, there was a randomly selected critical position (at 0^o^, 90^o^, 180^o^, or 270^o^). If infant was able to continually track stimulus for 10° preceding critical position, then 2nd stimulus, a 3D blue box, would appear in center of screen. The original smiley face stimulus continued to rotate on its path despite appearance of 2nd stimulus. If infant remained fixated on 1st stimulus (ie, smiley face), both stimuli remained on screen for 4 further rotations of 1st stimulus, then both disappeared, followed by new trial. If infant did not make box appear after 9 rotations of first stimulus, next trial began	Number of box (ie, 2nd stimulus) appearances Number of rotations preceding box Switch response time from first to 2nd stimulus	Rees et al. (2019)^64^
	Orienting toward and away from objects at various spatial locations	Infant orienting with attention	Central fixation appeared as bright yellow dynamic smiley face, accompanied by classical music. When infant gaze was fixed on central fixation, eye-tracking assessor pressed a key to advance to trial. Infant saw a 100 ms spatial precue depending on trial type. The task consisted of 3 experimental conditions (valid, invalid, and double cues) and 1 control condition (no cue). A black dot serves as a spatial precue preceding a randomly presented target image. The number and location of black dot presented depend on types of cue conditions. 100-ms blank screen. Infant was displayed a target image for 1000 ms, target images consisted of 96 colorful everyday objects, some familiar and others unfamiliar to children, such as fruits, balls, and baskets. In valid cue condition, both cue and target image appeared on same side, while in invalid cue condition, target image was presented on opposite side of cue. Double cue condition involved cues on both sides, with target image presented at spatial location of 1 of 2 cued locations. In baseline condition, no cue was presented, but target image still appeared on either left or right side of screen. Infants received up to 24 trials per trial type.	Response time Cue facilitation score Cue interference score	Prado et al. (2020)^62^, Stephenson et al. (2022)^61^, Bragg et al. (2023)^66^
	Orienting toward and away from objects at various spatial locations	Saccadic reaction time	After calibration, in each session, infant was presented (1) long social scene, followed by (2) 5 saccade targets, (3) short social scene, (4) 5 saccade targets, (5) long social scene, (6) 5 saccade targets, (7) short social scene, and (8) 5 saccade targets. Infants received 2 sessions (8 blocks of 5 saccade targets, 4 long and 4 short social scenes. Saccade targets: Targets were colorful cartoon animations of common objects (eg, fish, soccer ball). Targets were presented one at a time in center of screen, and each subsequent target in a new randomly chosen on-screen location 10° away. After appearing on screen animations remained paused at first frame and accompanying audio silenced until infant looked at animation or a 1-s wait period elapsed. The animation was subsequently played for 2500 ms with an accompanying sound. Social scenes: Stimuli were 5-15 s–video recordings of female models. In short social scene 5 s video, model looked directly at camera and greeted infant in infant’s natural language. In long social scene 9.5-15 s video, model looked at camera while enacting a short story or a song appropriate for infant’s age range. The videos were taken in various environments (eg, living room, kitchen).	Saccadic reaction time, calculated as time interval from onset of saccade target to first entry of gaze into area of saccade target. Analysis of eye-tracking data during social scene viewing had not been reported.	Leppänen et al. (2022)^55^, Rockers et al. (2023)^60^
	Orienting toward and away from objects at various spatial locations	Visual disengagement time	Infant watched a dynamic attention grabber on center of screen. After infant fixated on stimulus, stimulus started to spin to maintain infant attention. After 600-700 ms, peripheral stimulus was presented to left or right side of screen in 1 of 3 conditions: *baseline, gap,* and *overlap.* Baseline condition: Peripheral stimulus appeared and central stimulus disappeared simultaneously. Gap condition: Central stimulus disappeared 200 ms prior to appearance of peripheral stimulus, reducing competition for attention. Overlap condition: Central stimulus remained on screen, but stopped rotating when peripheral appeared, increasing competition for attention Peripheral stimulus remained on screen until gaze fixation was detected, or until 2000 ms had passed. It became animated, and engaging sounds were played. Infants received 15 trials in each condition.	Disengagement time, calculated as difference in saccadic reaction time between baseline and overlap conditions.	McCann et al. (2023)^65^

### Findings from Randomized Controlled Trials

Three studies evaluated nutrition exposures in children through randomized controlled trials. Prado et al.^62^ evaluated the intervention of providing one egg per day to infants, using the VPC task to assess infant memory and the IOWA task to assess attention. This study involved the same sample assessed by Bragg et al.^66^ The trial enrolled 660 children 6-9 months old in Malawi, with a 10% loss to follow-up rate. About 2/3 of the children were assessed using eye-tracking methods at the end of the intervention period when they were between 12 and 15 months old. No significant effects of the intervention on the eye-tracking–based tasks were observed. Similarly, the intervention did not significantly affect other developmental outcomes assessed by non–eye-tracking methods, such as the Malawi Developmental Assessment Tool (MDAT) and elicited imitation.

Similarly, another randomized controlled trial in Malawi by Stephenson et al^61^ used the same eye-tracking–based tasks (ie, the VPC and IOWA tasks) and found no effects of 3 different RUTF formulations (RUTF made with high-oleic [HO] peanuts and palm oil with added DHA [DHA-HO-RUTF] vs without added DHA [HO-RUTF] vs standard RUTF) among infants with MAM. Nonetheless, significant effects were observed on gross motor and social domain *z* scores assessed using the MDAT. It is worth noting that the intervention period lasted only until a clinical outcome was reached, or for a maximum of 6 bi-weekly follow-up visits. The study enrolled 2758 infants 6-59 months old. However, for the eye-tracking outcomes, a subset of participants was systematically selected for testing based on age at the time of assessment. Among those selected, around 60%-70% completed eye-tracking assessments and were included in the analysis (*n* = 380 in DHA-HO-RUTF vs *n* = 350 in HO-RUTF vs *n* = 402 in S-RUTF groups). Notably, this study did not include a group without intervention or healthy controls, nor did it perform eye-tracking assessments at baseline.

The most recent randomized controlled trial involving children that utilized eye trackers, conducted by Rockers et al.,^60^ evaluated a home visit intervention in South Africa aimed to improve child attention assessed using the SRT task. The intervention, which included nutrition counseling, began at birth and continued until the children reached 24 months of age, with additional follow-up until 36 months. Assessments were conducted at multiple time points (7, 17, and 36 months old), with the percentage of children assessed and analyzed varying across these time points (55.7%, 86.7%, and 82.6%, respectively). The home-visit intervention resulted in lower SRT at 7 and 17 months old, indicating faster attention shifting, but these differences were no longer present at 36 months old. This study did not observe any significant effects on developmental scores assessed using MDAT.

### Findings From Prospective Cohort and Cross-Sectional Studies

We identified 5 prospective cohort studies and 1 cross-sectional and prospective cohort study that utilized eye-tracking methods, with only 1 study assessing a prenatal exposure. Rees et al.^64^ conducted a prospective cohort study evaluating the effects of prenatal DHA intake during the 2nd and 3rd trimesters of pregnancy on infant memory, assessed using a habituation task, and attention, assessed using a visual attention task. Data on DHA intake was collected using the Diet History Questionnaire (DHQ; National Institute for Cancer [NIC], United States), which assesses diet over 3 months and includes an evaluation of dietary supplements. This study followed mothers in England from pregnancy until their children reached 9 months of age, with a relatively high proportion of children assessed (94 out of 107) despite the small sample size. However, no significant differences were observed on either task based on prenatal DHA intake during the 2nd and 3rd trimesters.

Cai et al.^63^ examined the influence of breastfeeding on the relational binding task, which indicates infant memory, and the visual expectation task depicting attention. This prospective cohort study followed pregnant women in Singapore until their children were 24 months old. Of the 1247 pregnant women enrolled, around 15% of children were assessed and included in the analysis at 6, 18, and 24 months old. Infants with high breastfeeding exposure—defined as those who were exclusively or predominantly breastfed until 4 months and continued with at least partial breastfeeding until the age of 6 months or beyond—showed better memory at 6 months on the relational binding task compared to those who were weaned and exclusively formula fed before 3 months. However, no other significant associations were identified in this study.

In a prospective cohort study in Malawi, anthropometric indicators of infant nutritional status, including LAZ and WAZ, were evaluated by Pyykkö et al.^49^ Infants were followed from the neonatal period until they were 9 months old. Eye-tracking–based tasks focusing on attention—comprising visual search, anticipatory attention shifts, and attention to faces—were performed at 9 months, with 86% of children assessed and analyzed out of 444 enrolled. However, neither LAZ nor WAZ at birth or at 9 months was associated with any attention markers at 9 months.

The status of MAM has been shown to influence eye-tracking results. Leppänen et al^55^ compared infants undergoing treatment for MAM in Sierra Leone with infants without MAM using the SRT task. This prospective cohort study enrolled 138 infants with MAM, defined by mid–upper arm circumference ≥11.5 cm and <12.5 cm without bipedal edema, and 60 age-matched controls 7-11 months old. Around 70% of the infants completed the SRT task. Infants with MAM demonstrated higher adjusted SRT at baseline, indicating slower attention shifting, but a significant improvement in SRT was observed after 4 weeks of treatment compared to controls.

Infant plasma choline, TMAO, and iron status were also significantly associated with eye-tracking–based tasks related to infant attention and memory. Bragg et al.^66^ conducted cross-sectional and cohort analyses of the randomized controlled trial in Malawi conducted by Prado et al.^62^ The data from 400 infants were examined at baseline, when they were 6 to 9 months old and at endline, when the infants were 12 to 15 months old. Higher plasma choline level was associated with slower IOWA response time, indicating slower attention shifting, but faster processing speed on the VPC task, indicating faster learning. Meanwhile, higher plasma TMAO level at baseline was associated with slower processing speed on the VPC task 6 months later. Iron status at 5 months old was evaluated by McCann et al.^65^ in Gambia. This cohort study assessed visual disengagement at multiple time points (5 months, 8 months, 12 months, 18 months, 24 months, and 3-5 years), with the percentage of children assessed and analyzed ranging from 41% to 65% of the 258 children enrolled. Significant differences in visual disengagement trajectories were found between infants in the highest and lowest terciles of soluble transferrin receptor levels at 5 months. However, no differences were observed based on ferritin and hemoglobin levels.

### Cross-Cultural Feasibility

Out of the 9 studies, only 2 were conducted in high-income countries. There was considerable variability in how studies reported the number of children assessed and included in the analysis for eye-tracking assessments. All studies conducted in low- and middle-income countries reported the number of children included in the analysis. In contrast, although both studies from high-income countries reported the number of children who attended the visit or were assessed, they either partially reported the number analyzed (ie, only for 1 out of 3 time points)^63^ or did not specify it at all (ie, for any time point).^64^ The proportion of enrolled children who were assessed and had sufficient usable data for at least 1 eye-tracking–based task ranged from 41% to 87%. An exception was 1 study in Singapore, which captured only 15% of enrolled participants.^63^ Among children assessed, data loss (ie, proportion without sufficient usable data for analysis) ranged from 2% to 44% across studies and time points. The criteria used to define usable data varied depending on the eye-tracking task (eg, children with trials showing at least 70% fixation on the central stimulus prior to attention shift,^49^ children with at least 10 valid SRTs per visit^55^^,^^60^) Moreover, some of the included studies made adaptions to the stimuli, such as using local faces, highlighting the potential for cross-cultural applicability.^49^^,^^55^^,^^62^ One study reported initial negative perceptions in a low-income community.^62^ However, a similar challenge was found in a high-income country, where participants expressed a lack of interest in having their child undergo neurocognitive assessments.^63^ Overall, these results suggest that eye-tracking assessments in nutrition studies are feasible across diverse settings, although data quality and reporting practices remain inconsistent.

## DISCUSSION

Despite the limited number of nutrition studies utilizing eye-tracking assessments, 5 of the 9 studies reviewed demonstrated significant relationships between maternal and child nutrition exposures and child cognitive outcomes, particularly in attention and memory domains. However, there was a heterogeneity in both the types of nutrition exposures and the eye-tracking assessments used, making it difficult to draw generalizable conclusions about the effects of early life nutrition exposures on child cognitive outcomes measured using eye trackers. Nutrition exposures associated with significant results included breastfeeding, supplementary feeding for MAM, home visit interventions, infant plasma choline, TMAO, and iron status.^55^^,^^60^^,^^63^^,^^65^^,^^66^

Eye-tracking methods allow for detecting subtle cognitive deficits or improvements when evaluating certain nutrition exposures. In contrast, global developmental assessments, which require more complex integration across domains, may underestimate these effects.^16^ However, in 1 of the included studies impacts on gross motor and social domains were observed when using global assessments (eg, MDAT), while no effects were found in the eye-tracking results.^61^ This result suggested that eye-tracking–based tasks complement global developmental assessments, particularly for evaluating attention and memory domains.

Furthermore, our scoping review showed that eye-tracking assessments are feasible across different settings. This finding aligns with a previous study comparing caregiver acceptance of the eye-tracking method in high-income and low-income countries, demonstrating high acceptance in both settings with comparable completion rates to traditional observational tests.^67^ A prior study comparing novelty preference scores using the VPC task among infants in the United States and rural Malawi also observed no significant differences, suggesting comparable performance across settings.^27^ However, more studies are needed to compare performance on other eye-tracking–based tasks across settings, because some cultural contexts may influence the results. For instance, infants in rural areas might be less familiar with looking at a computer screen, which could affect eye-tracking outcomes.

However, we noted several challenges in using eye-tracking assessments to evaluate impacts. Negative perceptions and lack of interest may occur among caregivers,^62^^,^^63^ which should be addressed through proactive community engagement and communication to describe the nature and purpose of the assessment according to the researchers or field teams. Because eye-tracking assessments may be relatively new in some areas, adequate training for local staff and piloting the assessments are crucial to ensure high-quality data.^46^^,^^56^ This approach can also help in detecting technical issues early on. Nonetheless, certain adjustments can be made to make the procedure easier for assessors, such as reducing the sampling rate to ease calibration.^46^ Researchers must also be cautious when transporting eye-tracking tools to remote areas with inadequate transportation infrastructure. One study conducted in a rural setting reported a software error,^62^ although the specific cause of the error was not further described.

We also identified considerable within-person variation in eye-tracking results over time. Hence, evaluating changes or improvements of the scores at the individual level may be less suitable than group-level evaluations (eg, intervention vs control groups) measured over time. For group-level comparisons, however, obtaining baseline assessments and including healthy infants as controls are crucial to confirm whether a nutrition intervention helps children catch up. For example, in 1 of the included studies comparing infants treated for MAM with healthy controls, the MAM-treated group had higher baseline SRT, but it was shortened after 4 weeks compared to controls, suggesting that the treatment may have helped the participans to catch up in reaction time.^55^ In our review, we also observed heterogeneity in the timing of assessments, which may influence the ability to detect group-based differences. Of note, the group-level evaluations are best performed during periods when differences are still potentially detectable using the same tasks. Otherwise, modified or alternative tasks that are more sensitive to differences in older children might be needed. For instance, preterm infants showed slower IOWA response time at 5 months compared to full-term infants, but these differences were no longer detectable at 10 months, suggesting a “catch-up.”^68^ Nonetheless, at 10 months, preterm infants exhibited attentional deficits specifically in tasks involving saccade canceling, shifting, and/or inhibition.^69^ Additionally, a minimum number of successful attempts may be required during assessments, which can lead to potential data loss. Inconsolable crying and lack of focus among children were identified as key challenges contributing to data loss, highlighting the need for an assessor experienced with children to address these issues.

We noted a few limitations of this scoping review. First, we limited our review to peer-reviewed, published studies to ensure methodological transparency and completeness of reported data; however, this approach may have introduced publication bias by excluding unpublished studies, conference abstracts, or registered clinical trials without published outcomes. The data extracted from the studies only allowed qualitative interpretation of the evidence due to the lack of uniformity across studies in nutrition exposures and types of eye-tracking–based tasks. Hence, in this review we were only able to summarize the current state of the field, and more nutrition studies utilizing eye-tracking assessments are warranted to identify which tasks are more sensitive to certain early life nutrition exposures. Furthermore, when selecting tasks, careful consideration should be given to those that are reliable and associated with long-term outcomes. Because nutrition exposures may interact with environmental factors in influencing cognition,^8^ future studies conducted in varied contexts and populations are also warranted. Last, although in our scoping review we aimed to include studies evaluating any cognitive domain using eye trackers, only attention and memory were represented among the included studies. Prior research that did not include nutrition exposures have used eye-tracking assessments to measure other cognitive domains, including language, social cognition, executive function, and predictive processing.^22–25^ These domains should be explored in future nutrition studies, particularly as our understanding of early cognitive precursors continues to grow and more eye-tracking tasks become available for evaluating development in early life.

Overall, our scoping review suggests that eye-tracking assessment is a promising method for evaluating child attention and memory in nutrition studies, with applicability across various settings. The strengths of this review included a comprehensive literature research, double title–abstract screening, full-text eligibility assessment, and data extraction. Although eye-tracking assessment is a feasible method for use in studies focusing on early-life nutrition, it is crucial to select appropriate eye-tracking–based tasks for a more sensitive evaluation of nutrition interventions.

## CONCLUSION

Eye-tracking assessment is a feasible and promising method for use in nutrition studies across settings. It enables the detection of subtle deficits or benefits of certain nutrition interventions, a challenge often faced with global developmental tests. However, the selection of eye-tracking–based tasks should be carefully tailored to the type of nutrition exposures, desired long-term outcomes, and easibility of proper training for local staff. Future studies are warranted to draw more generalizable conclusions about the impact of early life nutrition exposures on child cognitive outcomes measured using eye trackers.

## Supplementary Material

nuaf235_Supplementary_Data
