# Supplementary material for: Eyes on Development: Exploring the Impact of Early Life Nutrition Exposures on Child Cognition Through Eye-Tracking Assessment
Source: Nutr Rev. 2025 Dec 6;84(10):2193–212. doi: 10.1093/nutrit/nuaf235 (PMC13537450; doi:10.1093/nutrit/nuaf235)
Supplement: Supplementary file 1 [file nuaf235_supplementary_data.docx]

**Supplementary Materials**

**Table S1.** Search terms used for the scoping review

| **Database** | **Population** | **Intervention/Comparison** | **Outcome** |
| --- | --- | --- | --- |
| Pubmed | child OR infant OR toddler OR infancy OR pregnan* OR lactati* OR breastfe* | "Diet, Food, and Nutrition"[Mesh] OR "Nutrition Disorders"[Mesh] OR nutrition*[tiab] OR supplement*[tiab] OR food*[tiab] OR diet*[tiab] OR meal*[tiab] OR macronutrient*[tiab] OR micronutrient*[tiab] OR protein*[tiab] OR lipid*[tiab] OR omega*[tiab] OR polyunsaturated fatty acids[tiab] OR PUFA[tiab] OR "Fatty Acids, Omega-3"[Mesh] OR iron[tiab] OR zinc[tiab] OR iodine[tiab] OR copper[tiab] OR vitamin*[tiab] OR “Carotenoids”[Mesh] OR "Vitamin B 6"[Mesh] OR "Folic Acid"[Mesh] OR "Vitamin B 12"[Mesh] OR "Ascorbic Acid"[Mesh] OR "Vitamin D”[Mesh] OR choline[tiab] OR probiotic*[tiab] OR prebiotic*[tiab] OR protein*[tiab] OR selenium[tiab] | "Eye Movement Measurements"[Mesh:NoExp] OR "Eye-Tracking Technology"[Mesh] OR ("Visual Perception"[MeSH] AND (Cognition[Mesh] OR Attention[Mesh] OR Memory[Mesh])) OR “eye track*”[tiab] OR "eye-track*"[tiab] OR eyetrack*[tiab] OR “gaze track*”[tiab] OR "Gaze-Track*"[tiab] OR Gazetrack*[tiab] OR "eye movement track*"[tiab] OR “looking time”[tiab] OR “looking behavi*”[tiab] OR Fagan[tiab] OR habituation*[tiab] |
| Embase | child* OR infant* OR toddler* OR infancy OR pregnan* OR lactati* OR breastfe* | 'nutrition'/exp OR 'fatty acid'/exp OR 'nutritional disorder'/exp OR nutrition*:ab,ti,kw OR supplement*:ab,ti,kw OR food*:ab,ti,kw OR diet*:ab,ti,kw OR meal*:ab,ti,kw OR macronutrient*:ab,ti,kw OR micronutrient*:ab,ti,kw OR protein*:ab,ti,kw OR lipid*:ab,ti,kw OR omega*:ab,ti,kw OR polyunsaturated fatty acids:ab,ti,kw OR PUFA:ab,ti,kw OR iron:ab,ti,kw OR zinc:ab,ti,kw OR iodine:ab,ti,kw OR copper:ab,ti,kw OR vitamin*:ab,ti,kw OR 'carotenoid'/exp OR 'pyridoxine derivative'/exp OR 'folic acid derivative'/exp OR folate OR 'cobalamin derivative'/exp OR 'ascorbic acid'/exp OR 'vitamin D'/exp OR choline:ab,ti,kw OR probiotic*:ab,ti,kw OR prebiotic*:ab,ti,kw OR protein*:ab,ti,kw OR selenium:ab,ti,kw | 'eye movement monitor'/exp OR 'eye-tracking technology'/exp OR 'eye tracking'/exp OR ('vision'/exp AND 'cognition'/exp) OR “eye track*”:ab,ti,kw OR "eye-track*":ab,ti,kw OR eyetrack*:ab,ti,kw OR “gaze track*”:ab,ti,kw OR "Gaze-Track*":ab,ti,kw OR Gazetrack*:ab,ti,kw OR "eye movement track*":ab,ti,kw OR “looking time”:ab,ti,kw OR “looking behavi*”:ab,ti,kw OR Fagan:ab,ti,kw OR habituation*:ab,ti,kw |
| Food Science and Technology Abstracts | (child* or infant* or toddler* or infancy or pregnan* or lactati* or breastfe*).mp. | nutrition/ or malnutrition/ or omega-3 fatty acids/ or retinoids/ or vitamin b6/ or folates/ or folic acid/ or cobalamins/ or ascorbic acid/ or vitamin d/ or (nutrition* or supplement* or food* or diet* or meal* or macronutrient* or micronutrient* or protein* or lipid* or omega* or "polyunsaturated fatty acids" or pufa or iron or zinc or iodine or copper or vitamin* or choline or probiotic* or prebiotic* or protein or selenium).ab,ti. | (visual.mp. and (cogniti* or attenti* or memory or language or perception).ab,ti.) or ("eye movement" or "eye track*" or "eye-track*" or eyetrack* or "gaze track*" or "Gaze-Track*" or Gazetrack* or "eye movement track*" or "looking time" or "looking behavi*" or Fagan or habituation*).ab,ti. |
